# Cardiovascular risk profiles of GnRH agonists and antagonists: real-world analysis from UK general practice

**DOI:** 10.1007/s00345-020-03433-3

**Published:** 2020-09-26

**Authors:** Patrick Davey, Mike G. Kirby

**Affiliations:** 1 Northampton General Hospital, Cliftonville, Northampton, NN1 5BD UK; 2 Trends in Urology & Men’s Health, Baldock, Herts, SG7 5DX UK

**Keywords:** Prostate cancer, Degarelix, GnRH agonist, GnRH antagonist, Cardiovascular

## Abstract

**Purpose:**

Androgen deprivation therapy (ADT) is the mainstay for the management of metastatic prostate cancer. Available pharmaceutical ADTs include gonadotropin-releasing hormone (GnRH) agonists and antagonists. Here, real-world data are presented from the UK general practitioner Optimum Patient Care Research Database. The study investigated the hypothesis that GnRH antagonists have lower cardiac event rates than GnRH agonists.

**Methods:**

The incidence of cardiac events following initiation of GnRH antagonist or agonist therapy was investigated in a population-based cohort study conducted in UK primary care between 2010 and 2017.

**Results:**

Analysis of real-world data from the UK primary care setting showed that relative risk of experiencing cardiac events was significantly lower with degarelix, a GnRH antagonist, compared with GnRH agonists (risk ratio: 0.39 [95% confidence interval 0.191, 0.799]; *p* = 0.01). Patients that received degarelix as first-line treatment switched treatment more frequently (33.7%), often to a GnRH agonist, than those who initiated treatment with a GnRH agonist (6.7–18.6%).

**Conclusion:**

Screening for known or underlying vascular disease and identifying those at high risk of a cardiac event is important for risk mitigation in patients with prostate cancer receiving hormone therapy. The GnRH antagonist degarelix conferred a significantly lower risk of cardiac events than GnRH agonists. Prior to treatment, patients should be stratified based on level of cardiovascular (CV) risk, and appropriate lifestyle, and pharmacological interventions to mitigate CV risk should be recommended. CV risk factors and patient response to the intervention should be monitored at regular intervals.

## Introduction

Prostate cancer accounts for ~ 20% of new cancer diagnoses in the USA and the EU, and is now the most commonly diagnosed cancer in England [1–3]. In the UK, there were around 47,700 new annual cases of prostate cancer from 2014 to 2016, 49,029 new annual cases in 2018, and incidence rates are estimated to rise by 12% between 2014 and 2035, to 233 cases per 100,000 men by 2035 [3]. In 2017, prostate cancer accounted for 14% of all cancer deaths in men in the UK [4]. Data released by Public Health England in 2018 indicate that prostate cancer is now the most diagnosed cancer in England, with almost 8000 more diagnoses of prostate cancer in 2018 than the previous year [5].

Androgen deprivation therapy (ADT) is the mainstay for the management of metastatic prostate cancer, and surgical castration has been superseded by non-surgical therapies, which are now the standard of care [5–7]. Available pharmaceutical ADTs include gonadotropin-releasing hormone (GnRH) agonists, such as leuprolide, goserelin, triptorelin and buserelin acetate, and more recently, GnRH antagonists, such as degarelix and abarelix (approved in Germany and The Netherlands) [5, 8].

The incidence of cardiovascular disease (CVD) and prostate cancer increases with age, and CVD incidence and mortality are high in men with prostate cancer [9–11]. ADTs, and specifically GnRH agonists, have been associated with increased cardiovascular (CV) morbidity and mortality in observational studies, and men with pre-existing CVD are most at risk [12–17]. Data suggest that the risk for cardiac events might be lower with the GnRH antagonist degarelix, compared with GnRH agonists, particularly in patients with pre-existing CVD [18–21].

A recent analysis of real-world data from a UK general practitioner database comparing CV outcomes in patients treated with degarelix vs GnRH agonists is presented and discussed in this context.

## Methods

### Study design

A post hoc analysis of real-world data from the UK general practitioner database [Optimum Patient Care Research Database (OPCRD)] was conducted between 2010 and 2017. Over 700 general practices collaborate with OPC to contribute anonymized, patient-level diagnostic, clinical and prescribing information data to the OPCRD to enable scientific research to better understand chronic diseases and improve health outcomes [22]. To better understand if, in a real-world setting, GnRH antagonists have lower cardiac event rates than GnRH agonists, the database was used to compare cardiac event rates in patients with prostate cancer treated with degarelix vs GnRH agonists (leuprorelin, goserelin or triptorelin). Preliminary data were presented at the American Urological Association 2020 [23].

### Patients and baseline characteristics

UK primary care patients (*n* = 9081) with prostate cancer, aged ≥ 40 years, with no previous use of degarelix, leuprorelin, goserelin or triptorelin were included in analyses. Baseline characteristics (e.g. age, sex, body mass index, prostate-specific antigen [PSA] levels) were based on the data available closest to the initial prescription date. Information of pre-existing CVD and diabetes was based on data available up to the point of first ADT prescription. Pre-existing cardiac events were examined in a ‘baseline period’ of at least 1 year prior to the index prescription date (IPD, i.e. first prescription for degarelix or agonist).

### Study end points

The aims of the study were to investigate the incidence of cardiac events following initiation of GnRH antagonist (degarelix) or GnRH agonist (leuprorelin, goserelin or triptorelin) as therapy in patients with prostate cancer.

Following initiation of degarelix or GnRH agonist, the risk of cardiac events [heart failure; myocardial infarction (MI); arrhythmia; ischaemic heart disease (IHD); and all events combined (any cardiac)] for degarelix relative to each GnRH agonist was assessed. Patients were followed from the IPD up until the date the patient either died, transferred out of general practice, switched to second-line treatment, the date of cardiac event, or until the end of data collection, whichever event came first.

The time to switch from first- to second-line treatment was also determined. The date of the first prescription of second-line treatment was defined as the switching date. When there was a gap of 2–6 months before the switching date, the start date of the gap was defined as the switching date.

### Statistical analysis

Statistical analyses were conducted post hoc. Point estimation was calculated by maximum likelihood and the confidence intervals (CIs) were based on a Gaussian approximation on the logarithmic scale [24, 25]. *p* values (two-sided tests) were based on the same Gaussian assumptions as for the CIs. Differences between groups were assessed using Chi-square test, Fisher’s exact test, Kruskal–Wallis or analysis of variance test (when appropriate). The differences were considered significant if the *p* values were < 0.05. No multiplicity adjustments were performed, in line with the reasoning in Sect. 2.4 of Committee for Proprietary Medicinal Products Points to Consider on Multiplicity Issues in Clinical Trials [26]. R version 3.6.3 (2020-02-29) (R Core Team 2018) was used for all analyses. All the tests were performed with SAS software, version 9.3 (SAS Institute, Cary, NC).

## Results

### Study population

**Fig. 1:**
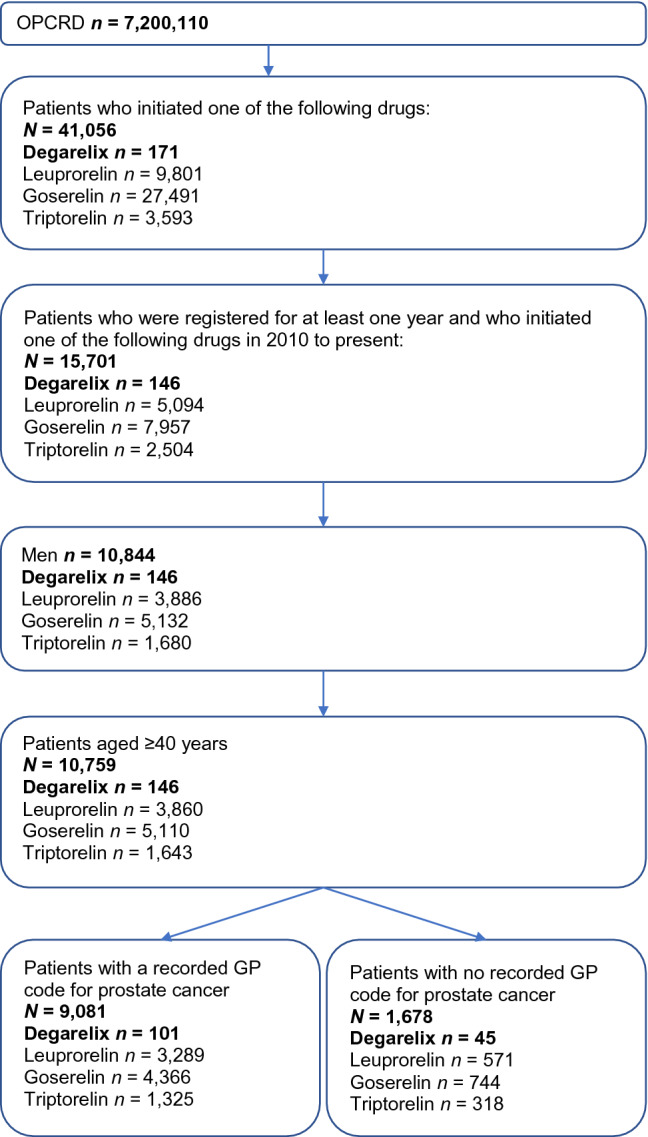
Consort diagram

In total, 101 patients received the GnRH antagonist degarelix, while the remainder of the cohort received a GnRH agonist (leuprorelin, *n* = 3289; goserelin, *n* = 4366; triptorelin, *n* = 1325) as first-line treatment (Fig. 1). Baseline and disease characteristics of patients with prostate cancer receiving each treatment are presented in Table 1. Overall, baseline characteristics appear comparable; however, prostate cancer severity and cardiac history between patients receiving degarelix vs GnRH agonists differed (in the degarelix group, PSA levels were higher and more patients had cardiac disease at baseline).

**Table 1:** Baseline characteristics of prostate cancer patients prescribed degarelix, leuprorelin, goserelin, or triptorelin in a UK population-based cohort study (*n* = 9081)

Baseline characteristics^a^	Degarelix users *n* = 101	Leuprorelin users *n* = 3289	Goserelin users *n* = 4366	Triptorelin users *n* = 1325
Age, year	*n* = 100	*n* = 3276	*n* = 4366	*n* = 1325
Mean (SD)	74.8 (9.0)	75.9 (8.6)	74.0 (8.5)	75.3 (8.3)
BMI, kg/m^2^, *n* (%)	*n* = 93	*n* = 3091	*n* = 4012	*n* = 1207
Mean (SD)	26.9 (5.0)	27.4 (4.9)	27.5 (4.5)	27.3 (4.5)
Overweight: 25–30	39 (41.9)	1364 (44.1)	1836 (45.8)	548 (45.4)
Obese: > 30	21 (22.6)	745 (24.1)	993 (24.7)	295 (24.4)
Smoking status, *n* (%)	*n* = 97	*n* = 3162	*n* = 4103	*n* = 1258
Current smoker	9 (9.3)	278 (8.8)	458 (11.2)	146 (11.6)
Ex-smoker	49 (50.5)	1464 (46.3)	1858 (45.3)	567 (45.1)
PSA, ng/ml, closest to baseline, *n* (%)	*n* = 67	*n* = 2663	*n* = 3260	*n* = 1115
Median (IQR)	72.4 (3.7–273.0)	10.0 (1.4–36.7)	8.0 (0.8–24.9)	10.6 (1.6–36.4)
< 20	27 (40.3)	1727 (64.9)	2312 (70.9)	694 (62.2)
≥ 20	40 (59.7)	936 (35.1)	948 (29.1)	421 (37.8)
Testosterone, ng/ml	*n* = 5	*n* = 240	*n* = 324	*n* = 91
Mean (SD)	14.7 (4.9)	16.2 (18.4)	13.8 (13.8)	15.4 (15.1)
Comorbidity ever before/at baseline, *n* (%)				
Cardiovascular disease	38 (37.6)	1075 (32.7)	1288 (29.5)	385 (29.1)
IHD	22 (21.8)	639 (19.4)	822 (18.8)	213 (16.1)
HF	4 (4.0)	168 (5.1)	154 (3.5)	53 (4.0)
MI	15 (14.8)	324 (9.8)	420 (9.6)	88 (6.6)
Arrhythmia	20 (19.8)	615 (18.7)	669 (15.3)	222 (16.7)
Chronic kidney disease	13 (12.9)	524 (15.9)	598 (13.7)	208 (15.7)
Hepatic impairment	2 (2.0)	85 (2.6)	121 (2.8)	39 (2.9)
Osteoporosis	2 (2.0)	64 (1.9)	94 (2.1)	21 (1.6)
Urticaria	2 (2.0)	88 (2.7)	152 (3.5)	30 (2.3)
UTIs				
1	9 (8.9)	169 (5.1)	229 (5.3)	65 (4.9)
2	2 (2.0)	25 (0.8)	54 (1.2)	
> 2	0 (0)	26 (0.8)	19 (0.4)	5 (0.4)
Diabetes mellitus	19 (18.8)	532 (16.2)	704 (16.1)	213 (16.1)
Drug use 6 months before/at baseline, *n* (%)				
Antithrombotic treatment	50 (49.5)	1297 (39.4)	1676 (38.4)	520 (39.2)
Anti-androgens	10 (9.9)	1185 (36.0)	1521 (34.8)	612 (46.2)

*BMI* body mass index, *HF* heart failure, *IHD* ischaemic heart disease, *IQR* interquartile range, *MI* myocardial infarction, *PSA* prostate-specific antigen, *SD* standard deviation, *UTI* urinary tract infection

^a^Closest to baseline for: age, sex, BMI, smoking status, PSA, testosterone; ever before baseline: comorbidity

### Cardiac events and other adverse events with degarelix vs GnRH agonists

**Fig. 2:**
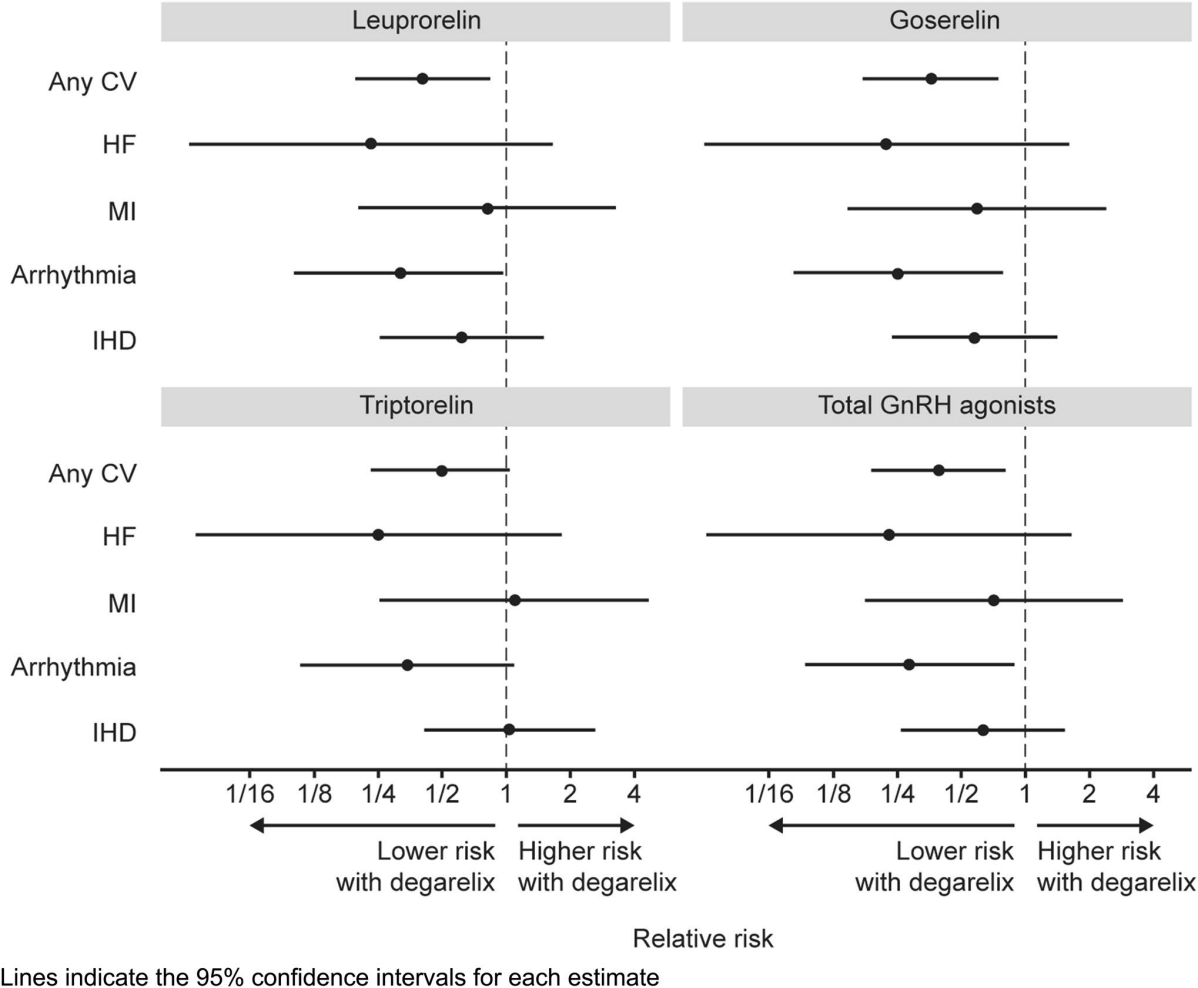
Estimated relative risk of experiencing cardiovascular events in patients with prostate cancer treated with first-line degarelix (*n* = 101) vs leuprorelin (*n* = 3289), goserelin (*n* = 4366), triptorelin (*n* = 1325), and pooled GnRH agonists (*n* = 9081)

The relative risk of experiencing any cardiac event was lower with degarelix than all GnRH agonists (risk ratio [RR] 6.9% vs 17.7%; 0.39 [95% CI 0.191, 0.799]; *p* = 0.01). This was significant vs leuprorelin (RR 0.40 [95% CI 0.197, 0.829]; *p* = 0.01) and goserelin (RR 0.36 [95% CI 0.175, 0.735]; *p* = 0.01), and nearly but not quite reaching statistical significance vs triptorelin (RR 0.50 [95% CI 0.240, 1.027]; *p* = 0.06). The effect of degarelix on cardiac event rates appeared to be largely driven by reductions in heart failure and arrythmia. By comparison, treatment effects on MI and IHD treatment were comparatively weak. The low number of patients in the degarelix group precluded any meaningful subgroup analyses (Table 2 and Fig. 2).

**Table 2:** Cardiovascular events in patients with prostate cancer treated with degarelix, leuprorelin, goserelin or triptorelin

	Degarelix users *n* = 101	Leuprorelin *n* = 3289	Goserelin *n* = 4366	Triptorelin *n* = 1325	GnRH agonists (pooled) *n* = 8980
Patients with an incident cardio event after initiation of therapy					
Any cardiac event, *n* (%)	7 (6.9)	564 (17.1)	844 (19.3)	185 (14.0)	1593 (17.7)
Relative risk (95% CI)^a^	–	0.40 (0.197, 0.829)	0.36 (0.175, 0.735)	0.50 (0.240, 1.027)	0.39 (0.191, 0.799)
*p* value	–	0.0135	0.0051	0.0590	0.0101
HF, *n* (%)	1 (1.0)	140 (4.3)	196 (4.5)	52 (3.9)	388 (4.3)
Relative risk (95% CI)^a^	–	0.23 (0.033, 1.646)	0.22 (0.031, 1.558)	0.25 (0.035, 1.806)	0.23 (0.033, 1.615)
*p* value	–	0.1441	0.1297	0.1703	0.1392
MI, *n* (%)	2 (2.0)	80 (2.4)	147 (3.4)	24 (1.8)	251 (2.8)
Relative risk (95% CI)^a^	–	0.81 (0.203, 3.266)	0.59 (0.148, 2.341)	1.09 (0.262, 4.560)	0.71 (0.179, 2.809)
*p* value	–	0.7717	0.4513	0.9026	0.6238
Arrhythmia, *n* (%)	3 (3.0)	308 (9.4)	516 (11.8)	115 (8.7)	939 (10.5)
Relative risk (95% CI)^a^	–	0.32 (0.104, 0.972)	0.25 (0.082, 0.768)	0.34 (0.111, 1.058)	0.28 (0.093, 0.867)
*p* value	–	0.0444	0.0154	0.0625	0.0271
IHD, *n* (%)	5 (4.9)	264 (8.0)	377 (8.6)	63 (4.7)	704 (7.8)
Relative risk (95% CI)^a^	–	0.62 (0.260, 1.461)	0.57 (0.243, 1.355)	1.04 (0.428, 2.530)	0.63 (0.268, 1.489)
*p* value	–	0.2720	0.2048	0.9290	0.2934
Time from initiation therapy to the first cardio event					
Mean, days (SD)	244.9 (220.1)	542.9 (572.0)	613.7 (618.5)	605.3 (502.3)	–
Median, days (IQR)	206.0 (85.0–219.0)	322.5 (137.0–751.0)	415.0 (136.5–880.5)	493.0 (215.0–853.0)	–

*CI* confidence interval, *CV* cardiovascular, *GnRH* gonadotropin-releasing hormone, *HF* heart failure, *IHD* ischaemic heart disease, *IQR* interquartile range, *MI* myocardial infarction, *SD* standard deviation

^a^Relative risk with degarelix vs leuprorelin, goserelin, triptorelin and pooled GnRH agonists

### Treatment switching from first-line ADT

Patients who received degarelix as first-line ADT switched treatment more frequently than those that received a GnRH agonist (33.7% vs 6.7–18.6%; Table 3). The mean time to switch was 289.8 (SD 261.5) days with degarelix. Most degarelix-treated patients who switched treatment did so after 3 months (67.6%), compared to within the first 3 months (32.4%) of first-line ADT, and most patients switched to leuprorelin (41.2%) or triptorelin (47.1%).

**Table 3:** First switch to second-line treatment in patients with prostate cancer prescribed first-line degarelix, leuprorelin, goserelin or triptorelin

Parameter	Degarelix users *n* = 101	Leuprorelin *n* = 3289	Goserelin *n* = 4366	Triptorelin *n* = 1325
Switched to second-line treatment				
*n* (%)	34 (33.7)	221 (6.7)	812 (18.6)	98 (7.4)
Time to switch from initiation therapy to second-line treatment, days				
Mean (SD)	289.8 (261.5)	453.6 (538.4)	559.1 (542.2)	354.2 (448.1)
Median (IQR)	234.0 (53.0–448.0)	280.0 (84.0–587.0)	378.0 (121.0–810.0)	167.0 (84.0–453.0)

*IQR* interquartile range, *SD* standard deviation

## Discussion

ADT lowers testosterone levels, which prevents androgen receptor signalling; however, GnRH agonists and antagonists suppress testosterone levels through different mechanisms, which may confer different CV risk as measured by event rates for patients with prostate cancer who receive ADT. GnRH agonists stimulate luteinizing hormone (LH) and follicle-stimulating hormone (FSH) release; this leads to reduced LH and FSH levels by receptor desensitization, resulting in suppressed testosterone secretion from the testicles [27–29]. In contrast, GnRH antagonists such as degarelix bind directly to GnRH receptors, leading to rapid suppression of LH and FSH and immediate decrease in testosterone secretion from the testicles, without an initial testosterone surge [30–32]. GnRH antagonists suppress FSH levels to a greater extent than GnRH agonists [33] and their differential effects on FSH levels might explain the differences in CV risk, development of atherosclerotic plaque formation, metabolic syndrome, and insulin resistance [34].

Treatment with GnRH agonists has been associated with increased CV morbidity and mortality [12, 13, 17, 35, 36]. The explanation of the differential CV risk between GnRH antagonists and agonists centres on T lymphocyte activation and destabilization of atherosclerotic plaques [37]. The GnRH receptor is expressed on pro-inflammatory T lymphocytes [38, 39] and upon activation an inflammatory cascade stimulates T cell proliferation, cytokine production, and macrophage activity leading to increased risk of atherosclerotic plaque rupture [20, 37], disruption of atheromatous plaques, and fatal coronary thrombi [40]. Unlike GnRH agonists, GnRH antagonists lack the ability to activate T lymphocytes, thus maintaining plaque stability [37].

Preclinical studies showed that treatment with leuprolide, but not with degarelix, induced atherosclerotic plaque instability and that degarelix was associated with reduced atherosclerosis, decreased adiposity, and reduced characteristics of metabolic syndrome, compared with leuprolide [41].

In the current study, real-world data from UK primary care settings was used to investigate whether the risk of cardiac events may be lower in patients receiving degarelix compared with GnRH agonists. The study found that the relative risk of experiencing any cardiac event was lower with degarelix than all GnRH agonists (*p* = 0.01). Differences in the RR were statistically significant for degarelix compared with leuprorelin and goserelin (both *p* = 0.01), but not for triptorelin (*p* > 0.05). The effect of degarelix on cardiac events appeared to be largely driven by reductions in heart failure and arrythmia. It should be noted that the number of patients taking degarelix in the UK is low, with clinicians currently using GnRH agonists as first-line treatments. However, the preliminary results of this study support findings from several other studies that suggest GnRH antagonists are associated with a lower cardiac risk than agonists.

Most but not all real-world observational data suggest that treatment with GnRH antagonists is associated with fewer cardiovascular event rates than treatment with GnRH agonists [35]. Real-world observational data from 2382 patients with advanced prostate cancer from a German claims database showed no significant differences in the incidence of CVD or diabetes between GnRH agonists or antagonists overall, although there was a significant increase in hypertension in patients receiving a GnRH agonist (16.4%) compared with those receiving a GnRH antagonist (6.9%, *p* = 0.022) [42]. Similarly, data from 35,118 ADT users identified from a French population-based claims reimbursement database demonstrated no significant association between GnRH antagonists and CV risk [43]. Finally, a retrospective analysis from the Scottish Cancer Registry reported a 30% and 50% increase in CVD risk with GnRH agonists and degarelix, respectively, compared with untreated patients. However, the authors state the association of higher CV risk with ADT compared with untreated patients was largely driven by CV events in GnRH agonist-treated patients [44].

In contrast and in line with the results from this study, an Italian real-world analysis showed the incidence of CV events was significantly higher in patients treated with GnRH agonists compared with antagonists (6.2 vs 8.8 per 100 person-year, *p* = 0.002) [45]. Further, disproportionality analysis from the pharmacovigilance database, Vigibase, found that of 10,504 and 1606 adverse drug reactions reported for GnRH agonists and antagonists, respectively, the respective numbers of cardiac-related events were 805 (7.7%) and 102 (6.4%). The time from initiation of therapy to onset of adverse event was over 1 year (mean 541.9 days, SD 909.6) [36].

There is conflicting data from randomized trials concerning cardiovascular event rates in GnRH agonist vs antagonist therapy. However, most of these trials were not set up to determine cardiovascular outcomes. One trial that was specifically set up to look at cardiovascular outcomes in (*n* = 80) those with pre-existing cardiovascular disease, showed GnRH antagonist treatment was associated with fewer major CV and cerebrovascular events than treatment with GnRH agonists. The absolute risk reduction for a major CV event (defined as either death, MI, cerebrovascular event, or percutaneous-angioplasty with coronary stent insertion) at 12 months using a GnRH antagonist was 18.1% (95% CI 4.6–31.2, *p* = 0.032) [19]. Further, a post hoc analysis of six Phase 3 trials (*n* = 2328) reported a significantly lower 1-year risk of cardiac events (arterial embolic and thrombotic events, haemorrhagic or ischaemic cerebrovascular conditions, MI, or other IHD) among men with pre-existing CVD initiating degarelix compared with GnRH agonist therapy. The absolute risk reduction during the first year was 8.2% and the number of patients needed to be treated to avoid one CV event or death was 12 [18].

The findings of the current study are also in line with a recent Phase 3 trial which investigated CV risk as a pre-specified safety analysis in 622 patients with advanced prostate cancer who received a GnRH antagonist (relugolix) compared with 308 patients who received a GnRH agonist (leuprolide), and suggested reduced CV risks with GnRH antagonists compared with agonists [46]. Among all patients, the incidence of major adverse CV events (defined as non-fatal MI, non-fatal stroke, and death from any cause) was 2.9% (exact 95% CI 1.7–4.5) in the relugolix group and 6.2% (exact 95% CI 3.8–9.5) in the leuprolide group (hazard ratio 0.46; 95% CI 0.24–0.88). Kaplan–Meier estimates of the major CV event incidence rate were consistent with a 54% lower risk in patients who received relugolix vs leuprolide (hazard ratio 0.46; 95% CI 0.24–0.88). In a subgroup of patients with a reported medical history of CV events, the incidence of major adverse CV events was 3.6% in the relugolix group vs 17.8% in the leuprolide group.

In the current study, more patients who were treated with degarelix as first-line treatment switched treatment than those who received first-line treatment with a GnRH agonist. Most patients switched either within the first 3 months of first-line treatment or after 12 months, and most switched to leuprorelin or triptorelin. Therapeutic drug switching does occur and may be done for convenience, to reduce cost or to reduce side effects [47]. However, while there is some evidence of clinical benefit in switching a patient from a GnRH agonist to an antagonist [48] there is limited evidence for the opposite approach with no established protocols for switching between these different classes of drug [47]. Further, with real-world evidence studies showing mean time to CV event beyond 1-year [36], long-term treatment on an antagonist may be required to reduce CV risk.

In summary, the available data suggests that GnRH agonists increase CV events, and this effect appears to be greatest in patients with pre-existing CVD, and GnRH antagonists may be associated with lower rates of certain CV events vs GnRH agonists, particularly in patients with pre-existing CVD. The results in the present study support the hypothesis that the risk of cardiac events may be lower in patients receiving a GnRH antagonist such as degarelix, compared with GnRH agonists. However, there are several important limitations to consider.

### Study limitations

All observational studies are vulnerable to bias and confounding, especially in terms of patient selection, differences in data capture and reporting. Indeed, an important limitation of the current study is that no steps were taken to minimize the risk of selection bias. Since some important differences in the baseline characteristics of patients were noted, including that more patients receiving degarelix had pre-existing CVD compared with patients receiving GnRH agonists, the current study results are vulnerable to confounding in terms of patient selection.

A further potential limitation is that the patients were all from a UK database, which could limit the generalizability of the findings to other regions. A major limitation was the small sample size of degarelix-treated patients, which prevented subgroup analysis of individual risk factors. The small sample size is common to other retrospective analyses of degarelix, with the low number of patients taking degarelix possibly due to clinicians favouring GnRH agonists as first-line treatments [42, 47]. Further, events of hypertension, stroke, and CV death were not included in the analysis. The higher CV risk associated with ADT was largely driven by cardiac events in GnRH agonist-treated patients.

Importantly, the statistical analyses were conducted post hoc and were not powered to determine statistical significance between therapies; in addition to the degarelix-treated patient group being small (*n* = 101), the stage of prostate cancer was unknown, PSA and testosterone levels were not available post-treatment, and data were from the OPCRD database; therefore, these findings may not be directly transferable to secondary care. Time from treatment initiation to first CV event was shorter with degarelix compared with GnRH agonists (244.9 days vs 542.9 for leuprorelin, 613.7 for goserelin and 605.3 days for triptorelin) which might reflect a greater cardiac event risk in these patients. A key limitation is that time on treatment was not collected as per the study protocol, and potential differences in exposure time between treatments could therefore not be corrected for. Further research is required on the association between degarelix and CV events; a randomized controlled trial administering degarelix or leuprorelin is currently ongoing [49].

## Conclusion

Although the recent UK real-word study had several important limitations, the data showed that the relative risk of cardiac events was significantly lower with degarelix as first-line ADT (specifically heart failure and arrythmia) compared with GnRH agonists. While not definitive, the literature has previously shown that GnRH agonist treatment increases CVD and diabetes risk and that negative effects may be greatest in patients with pre-existing CVD. In contrast, it appears that GnRH antagonist therapy in this study and others is associated with lower rates of CVD than in those treated with GnRH agonists.

Given these findings, screening for known and undiagnosed metabolic and CV risk factors may be the key to risk mitigation in patients with prostate cancer receiving ADT. Appropriate therapy for prostate cancer, lifestyle, and other pharmacological interventions should be recommended, and CV risk factors should be monitored regularly.
