# Correction to: Cardiovascular risk profiles of GnRH agonists and antagonists: real-world analysis from UK general practice

**DOI:** 10.1007/s00345-022-04032-0

**Published:** 2022-05-07

**Authors:** Patrick Davey, Mike G. Kirby

**Affiliations:** 1 Northampton General Hospital, Cliftonville, Northampton, NN1 5BD UK; 2 Trends in Urology & Men’s Health, Baldock, Herts, SG7 5DX UK

## Correction to: World Journal of Urology (2021) 39:307–315 10.1007/s00345-020-03433-3

In the original publication of the article, the authors omitted to disclose the following statement:

**Disclosure**

The design and analysis of the study reported in this article was entirely undertaken by Prof. David Price^3^, Hilda de Jong^4^ and Dr. Rupert Jones^5^ (please, see below for affiliations). We, the authors of the original publication did not participate in that research, but were involved in the interpretation for the article.

^3^Observational and Pragmatic Research Institute, Singapore, Singapore; Optimum Patient Care, Cambridge, United Kingdom; Centre of Academic Primary Care, Division of Applied Health Sciences, University of Aberdeen, Aberdeen, United Kingdom. Electronic address: dprice@opri.sg.

^4^Observational and Pragmatic Research Institute Pte Ltd (OPRI), Singapore.

^5^The Peninsula College of Medicine and Dentistry, Plymouth, UK.

